# The Quality of Medicines Used in Children and Supplied by Private Pharmaceutical Wholesalers in Kinshasa, Democratic Republic of Congo: A Prospective Survey

**DOI:** 10.4269/ajtmh.17-0732

**Published:** 2018-01-08

**Authors:** Benedetta Schiavetti, Evelien Wynendaele, Bart De Spiegeleer, Geremie J. Mbinze, Nicodème Kalenda, Roland Marini, Vera Melotte, Epco Hasker, Bruno Meessen, Raffaella Ravinetto, Josiane Van der Elst, Daniel Mutolo Ngeleka

**Affiliations:** 1 Department of Public Health, Institute of Tropical Medicine, Antwerp, Belgium;; 2 Drug Quality and Registration (DruQuaR) Group, Faculty of Pharmaceutical Sciences, Ghent University, Ghent, Belgium;; 3 Faculty of Pharmaceutical Sciences, Laboratory of Drug Analysis, University of Kinshasa, Kinshasa, Democratic Republic of Congo;; 4 Department of Pharmacy, CIRM, Laboratory of Analytical Chemistry, University of Liege (ULg), Liege, Belgium;; 5 Belgian Technical Cooperation (BTC), Bruxelles, Belgium;; 6 Belgian Federal Agency for Medicines and Health Products (FAGG-AFMPS), Bruxelles, Belgium;; 7 Direction de la Pharmacie et du Médicament (DPM), Kinshasa, Democratic Republic of Congo

## Abstract

Poor-quality medicines are a threat to public health in many low- and middle-income countries, and prospective surveys are needed to inform corrective actions. Therefore, we conducted a cross-sectional survey on a sample of products used for children and available in the private market in Kinshasa, Democratic Republic Congo: amoxicillin (AX) and artemether/lumefantrine (AL), powders for suspension, and paracetamol (PC) tablets 500 mg. Overall, 417 products were covertly purchased from 61 wholesalers. To obtain a representative sample, the products were weighted on their market shares and a subset of 239 samples was randomly extracted to undergo in-depth visual inspection locally, and they were chemically assessed at two accredited laboratories in Belgium. Samples were defined of “poor-quality” if they failed to comply with at least one specification of the International Pharmacopoeia (for AL) or United States Pharmacopoeia 37 (for AX and PC). Results are reported according to the Medicine Quality Assessment Reporting Guideline. The visual inspection detected nonconformities in the aspects of antimalarial powders for suspension, and poor-quality labels across all medicine types. According to chemical analysis, 27.2% samples were of poor quality and 59.5% of AL samples were underdosed in artemether. Poor quality was more frequent for locally manufactured antimalarials (83.3%, *P* = 0.021; 86.4%, *P* = 0.022) and PC (4.8%, *P* = 0.000). The poor quality of the surveyed products may decrease the treatment’s efficacy and favor the development of resistances to antimalarials. It is hoped that these findings may guide the corrective actions of the Democratic Republic of Congo Regulatory Authority, which was the main partner in the research.

## INTRODUCTION

There is growing evidence that poor-quality medicines represent an important problem,^1–7^ which may cause therapeutic inefficacy and prolonged illness or death,^8^ toxicity,^9^ increased drug resistances,^10–12^ as well as loss of trust in health-care systems. They are particularly prevalent in low- and middle-income countries (LMICs).^13–15^ Although the interpretation of existing data is blurred by the lack of harmonization in the methodologies and definitions used,^16^ two recent reviews suggested respectively that among antimalarials tested over a span of 67 years, 30.1% were of poor quality^16^ and that the rate of poor-quality medicines in sub-Saharan Africa ranges from 12% to 48%.^17^ The global supply chain is increasingly complex^18^ and the price competition may foster the trade of non-quality–assured products, whereas the under-resourced national medicines regulatory authorities (NMRAs) struggle to control their markets.^19–21^ Children in LMICs have often been the victims of poor-quality medicines^22,23^; a recent modeling study estimates that 3.75% of all malaria deaths in children aged less than 5 years in sub-Saharan Africa are due to poor-quality antimalarials.^24^ However, few studies have formally assessed the quality of pediatric formulations.^25^

The Democratic Republic of Congo (DRC) ranks 9th in the world for the mortality rate of children aged less than 5 years.^26^ Decades of public health sector’s dereliction led to the development of a huge informal health sector.^27^ Thus, the government included among the targets of the 2011–2015 National Health Development Plan the reduction of children mortality and the quality assurance of medicines.^28^ By the year 2015, 80% of the products on the market should have been of assured quality. Unfortunately, the achievement of this target cannot be measured as, beside specific case reports^29^ or surveys with small sample sizes,^30–34^ there is to date no sufficient evidence from surveillance and research. To the best of our knowledge, only another prospective survey carried out in DRC investigated the quality of oral solid dosage forms containing artemether (two samples only, both found to be compliant with the stated specifications).^30^ Other studies tested other antimalarials, antiretrovirals, and medicines for tuberculosis. Therefore, we conducted a survey on the quality of selected medicines used in children, to estimate the rate of poor-quality products in the formal private market in the capital city Kinshasa and to identify the possible risk factors. The survey was conducted in partnership with the NMRA, that is, the *Direction de la Pharmacie et du Médicament* (DPM).

## MATERIALS AND METHODS

The definitions used in this survey are provided in Box 1.

Box 1 **Definitions**

On 29 May 2017, delegates at the World Health Assembly have reached new agreement on substandard and falsified medical products. The new definitions are as follows^60^:

 *Substandards*: also called “out of specification,” these are authorized medical products that fail to meet either quality standards or their specifications, or both;

 *Falsified*: medical products deliberately/fraudulently misrepresented with respect to their identity, composition and/or source.

 *Unregistered or unlicensed medical products*: have not been assessed or approved by the relevant national or regional regulatory authority for the market in which they are marketed, distributed or used.

In addition to the World Health Organization definitions, the term *degraded* has been proposed for medicines that deteriorate because of poor storage or distribution conditions.^61^

**In this survey, we use the inclusive term “*poor-quality medicines*” because all these products have equally harmful consequences**^62,63^, **the analytical techniques used in this survey do not allow screening and validating falsified samples**^64^, and **this inclusive classification has been proposed by other authors and used in other published surveys.**

Definitions agreed by the study team for the purpose of this survey:

 a *medicine* is an active pharmaceutical ingredient (API) in a given pharmaceutical form, named by its international nonproprietary name (e.g., paracetamol 500 mg, tablets);

 a *product* is a medicine, in a finished pharmaceutical form, carrying a brand or generic name (i.e., the same medicine sold with the same brand but in two different dosages is considered as two different products);

 a *sample* is a group of physically identical products purchased in the same wholesaler (with the same generic or brand name, API, strength, dosage form, expiry date, manufacturer and batch number);

 a *product/wholesaler* is a product sold by a specific wholesalers. The same product sold by two different wholesalers is counted as two product/wholesaler.

 a *unit* within a sample is one bottle in case of oral solutions, and one blister in case of tablets.

 the *volume of distribution* is the amount of product sold over a period of time by one wholesaler.

Medicines were selected together with the DPM, based on eight criteria relevant for public health (Table 1). Artemether/lumefantrine (AL) 15 mg + 90 mg/5 mL, powder for suspension; amoxicillin (AX) 125 mg/5 mL or 250 mg/5 mL, powder for suspension; and paracetamol (PC) 500 mg, tablets are first-line treatments in DRC for malaria, acute respiratory infections, and fever, that is, the main causes of morbidity in children aged less than 5 years in the country.^28^ The specifics formulations were chosen after informal open interviews with key informants (general practitioners, pediatricians, and pharmacists). For PC, they reported that mothers and prescribers favor 500 mg tablets over pediatric formulations because they can be split and shared among family members. For AX and antimalarials, they reported that mothers and prescribers prefer powders for suspension because of ease of administration.

**Table 1:** Criteria for the selection of the medicines

	Amoxicillin	Artemether/lumefantrine	Paracetamol
1. They are first-line treatments for diseases with the highest pediatric morbidity rate in DRC (malaria, respiratory tract infections, and fever)	✓	✓	✓
2. The targeted diseases are included in the DRC National Health Development Plan	✓	✓	✓
3. They are included in a DRC national treatment protocol	✓	✓	✓
4. They are included in the last published WHO essential medicines list for children	✓	–	✓
5. They are included in the last published national essential medicines list	✓	–	✓
6. They are included in a reference pharmacopoeia (USP,* BP,† Int.Ph.‡)	✓	✓	✓
7. They cause toxicity or drug resistance if used in poor-quality formulations	✓	✓	✓

DRC = Democratic Republic of Congo; WHO = World Health Organization.

* United States Pharmacopoeia.

† British Pharmacopoeia.

‡ International Pharmacopoeia.

Kinshasa was selected as the study area because it is the largest urban zone in DRC,^35^ the main hub for importation and trade, and because of logistic accessibility and organizational ease.

Concerning the market sectors, the World Health Organization (WHO) distinguishes three levels^34^: 1) importers, central medical stores, and manufactures; 2) wholesalers, retailers, hospital clinics, and health centers; and 3) informal sector. We chose to survey the licensed wholesalers (level 2) because they play a central role in DRC by supplying the public, private, private not-for-profit, and confessional sectors all over the country, and because determining the prevalence of poor-quality medicines in the formal sector and upstream in the supply chain would allow making hypothesis about the downstream supply chain and the unregulated informal sector.^17^ On the one hand, we assume that rates of poor-quality medicines downstream in the distribution chain would be higher than those found in our survey, because of poorer storage conditions and less-frequent/weaker regulatory oversight at peripheral level. On the other hand, we assume that rates of poor-quality medicines in the informal sector would be higher than those found in our survey because of complete lack of any regulatory control and of higher likelihood of procurement from nonsecured suppliers. Among the 91 wholesalers included in the 2013 DPM list of licensed wholesalers, 80 were eligible (the other 11 had ceased their activity).

We conducted a cross-sectional survey based on standard operating procedures developed on the results of a pilot study conducted in December 2013 (B. Schiavetti. *Rapport de mission. Récolte d’information et achat pilote. Kinshasa, Décembre 2, 2013*. Available on request). The Medicine Quality Assessment Reporting Guideline (MEDQUARG) inspired the sampling strategy^36^: for example, the choice of the random sampling technique, the choice of referenced semantic (poor-quality, falsified, substandard, and degraded medicines); the choice of the collectors, timing, and location of the sampling; and the choice to submit the survey to the relevant Ethics Committees. A predefined quantity of all products available on the days of the collection at the 80 licensed wholesalers were purchased; a random sample based on the volume of distribution of each product in the market underwent visual inspection and chemical analysis.

Given an estimated range of 12–48% poor-quality medicines, the sample size was calculated considering that a proportion of 34% in our parent population would be a realistic premise. To obtain a precision of ±10% in the results and assuming equal distribution volumes, a sample size of 80 for each of the three selected medicines was calculated with the inference formula (95% confidence interval [CI]: *p* ± 1.96 √*pq*/*n*; CI, hypothetical proportion of nonconform samples [*p*], *q* = [*p* − 1], sample size [*n*]), thus a total of 240 samples.

Before the purchase, and to reduce the risk of collecting ineligible products (e.g., wrong formulation), eight physicians from the Public Health School of Kinshasa were trained as mystery shoppers.^7^ All survey’s staff were blinded to the purpose of the study. In DRC, wholesalers can only sell to health facilities, dispensaries, and private pharmacies.^37–39^ Thus, mystery shoppers were instructed to act as an employee of a pediatric practice in a rural area and to show a list of medicines explaining that “…we want to purchase a variety of products containing these medicines in order to evaluate the patients’ preferences and then file a bigger order.” To collect the largest possible variety of products, the mystery shoppers further asked “Do you have other products? Cheaper ones?” To perform the chemical analyses, minimal quantities to be purchased were predefined: six bottles of powders for suspension for AL, four bottles for AX, and 20 tablets for PC. The purchased samples were brought daily to the study center, where the following information was recorded: identification number, international nonproprietary name (INN), brand name, dosage, pharmaceutical form, batch number, stated manufacturer, purchase date, manufacturing date, expiry date, name of the wholesaler, number of units purchased, and unit price. The samples were stored at controlled environmental conditions according to WHO (max. 30°C; max. 65% relative humidity).^40^ On May 30, 2014, after completing the first round of visual inspection, the samples were transferred by airfreight to the Belgian Federal Agency for Medicines and Health Products (Brussels, Belgium).

After the purchase, the DPM requested the wholesalers to provide information about the units of product sold (volume of distribution) over two consecutive periods of time: January–December 2013 and January–April 2014. To extract 80 samples for each medicine type, each sample was allocated a weight, which was equal to its volume of distribution, divided by the volume of distribution of the sample with the smallest volume. Then, for each of the three medicines, we made a list where one sample had a weight of 1 and all the others had higher weights and occurred as many times as their weights. From these, we took random samples of 80 samples for each medicine multiple times and always with restitution. This process was continued until 80 different samples had been selected. After performance of the chemical analyses, the result of each sample was allocated an analysis weight equal to the number of times the sample had been selected during the sampling. The analysis with weight allowed us to make optimal estimates of the proportion of poor-quality products in the market, rather than of the proportion in our sample, and reflects whether poor-quality products have high or low market distribution.

Each sample was visually inspected twice: first in Kinshasa, by the inspectors of the DPM and then in Brussels, by the analysts of the quality control laboratories. They all used a standard checklist, jointly developed with the DPM, which includes 68 attributes of the primary (inner) and secondary (outer) packaging, related to six items: 1) physical appearance, 2) storage conditions, 3) identification, 4) traceability, 5) readability of the label/leaflet, and 6) leaflet contents (see Supplemental Material). The packaging quality attributes were defined accordingly to the national regulatory requirements. In case complete references were lacking, we referred to the International Pharmacopoeia (Int.Ph.)^41^ and to relevant WHO documents.^32,34,42,43^ The DPM inspectors recorded the findings in a checklist as yes/no answers; uncertainties were counterchecked by a second inspector and discussed within the team. The checklists were transferred to Belgium with the corresponding samples and reviewed by the analysts of the laboratories who carried out the second visual inspection before the chemical analyses.

The chemical analyses were conducted by two Belgian accredited laboratories: the Drug Quality and Registration Group of the University of Ghent tested the antimalarials and the Laboratory of Analytical Chemistry of Liège tested AX and PC samples. The following attributes were tested for AL for conformity with the requirements of the Int.Ph.† (Table 2): identity, dosage content, and deliverable volume. For AX and PC, the United States Pharmacopoeia (USP) 37^44^ was used as a reference for the following attributes: identity and dosage content, deliverable volume and pH (AX only), and mass variation (PC only) (Table 2). The dosage content was tested in duplicate, and the final result was calculated as the mean of the two values. For each out-of-specifications result, analyses were repeated and the result retained if the difference between the two sets of analyses (Δ) was ≤ 5%. In case of high variability between the results of the 1st and the 2nd set of analyses (Δ > 5%), a 3rd analysis was performed. The final value is the mean of non-deviating values. The chemical analysis could not screen for falsification or degradation.^45^

**Table 2:** Quality control analyses retained for the study and related specifications

Attributes	Artemether/lumefantrine	Amoxicillin	Paracetamol
Identity			
Retention times of the samples (*t*_Rs_) and of the standard (*t*_RS_)	*t*_Rs_ = *t*_RS_ (±5′)	*t*_Rs_ = *t*_RS_ (±5′)	*t*_Rs_ = *t*_RS_ (±5′)
Ultraviolet-Vis spectra	Superimposable	Superimposable	Superimposable
Content	90–110% l.c.	90–120% l.c.	90–110% l.c.
Mass uniformity*	n.a.	n.a.	AV ≤ 15%
Deliverable volume	90–110% l.c.	NLT 90% l.c.	n.a.
pH of the reconstituted solution	n.a.	5.0–7.5	n.a.

AV = acceptance value; l.c. = label claim; n.a. = not applicable; NLT = not less than.

* Uniformity of dosage unit was applied.

The data from sampling and visual inspection were double-entered into the EpiInfo^**®**^ database (version 3.5.4) by two pairs of trained data clerks in Kinshasa. Data quality and consistency were checked with data tables compare and validation functions. The results of the chemical analysis were entered in the same database by the survey coordinator on reception of the certificates of analysis from the laboratories. Data quality and consistency were further checked, before the statistical analysis, on a random dataset. Statistical analyses were performed with STATA^**®**^ (Stata Corp., College Station, TX) and EpiInfo^®^ (Center for Disease Control and Prevention, Atlanta, GA). The rate of poor-quality medicines was calculated first as crude proportion (non-weighted) and then as proportion weighted on the volume of distribution in the local market. The Fisher test was used to detect significant differences between groups in the risk factors analysis (*P* ≤ 0.05).

The ethical challenges of surveys on medicines’ quality have been analyzed in a recent publication.^46^ In this survey, the quantity of samples’ units to be collected was agreed with the DPM to avoid a negative impact on the local availability of medicines for patients. The accuracy of results was ensured by analyzing the samples in laboratories approved by a stringent NMRA and by testing each out-of-specifications result until confirmation. To protect the identity of the mystery shoppers, their names have not been mentioned in survey reports and publications; survey staff have signed nondisclosure agreements. The survey has been conducted in partnership with the DPM and the results were first reported to them. This survey was approved by the Institutional Review Board of the Institute of Tropical Medicine Antwerp (ref. 927/14) and by the Ethics Committee of the School of Public Health of the University of Kinshasa (ref. ESP/CE/005/2014).

## RESULTS

### Sampling.

Between April 7 and 18, 2014, 417 samples were collected from 61 of 80 eligible wholesalers (76.3%). Of the remaining wholesalers, 12 did not sell the target medicines, five had ceased activity, and two only sold to other wholesalers. Of the 417 samples, 47 were excluded because purchased units were insufficient and two because of wrong formulation (effervescent PC tablets). Of the remaining 368 samples, 165 were pediatric powders for suspension containing AL, 120 pediatric powders for suspension containing AX, and 83 PC tablets.

Between May 20 and 26, the DPM inspectors collected the standard forms filled in by the wholesalers with the volumes of distribution of each product. Two wholesalers did not provide the data. Data completeness was higher for the first trimester of 2014 (83% samples) than for 2013 (65% samples). Thus, the data of 2014 were used for extracting the final sample. Of the 373 products/wholesalers, data were missing for 25. For 38 products/wholesaler, data were provided by INN, without distinction among products. To deal with the missing information (*N* = 63), predictive values were calculated through a matrix: the ranks of the products per volume of distribution in the *x* axis and the ranks of the wholesalers in the market (provided by the DPM) in the *y* axis. Each missing value was calculated as the mean between the lower and the upper figure. The 240 samples for the analyses were extracted with STATA by random sampling weighted by the volume of distribution. One AL sample expired before analysis, and the final study population was of 79 AL, 80 AX, and 80 PC samples.

### Characteristics of the samples.

The origin of each sample was defined based on the manufacturer’s address stated in the label: 20.5% were labeled as manufactured in India, 19.3% in DRC, 14.2% in France, 11.7% in China, 4.6% in the Netherlands, 0.4% in Morocco, and 0.4% in Senegal. For the remaining 28.9%, the manufacturer’s address did not appear in the label (not defined, n.d.), but the DPM confirmed that they had been manufactured in DRC. Thus, the percentage of domestic samples rises to 48.2%.

The distribution by country of origin varied across medicines groups: 53.2% of AL were from India, whereas most AX and PC were locally manufactured (57.5% and 55.1%, respectively), 42.5% AX came from two French manufacturers, and 33.8% PC came from one Chinese manufacturer (Table 4). On average, 3.5 different batch numbers were collected for each product (min. 1; max. 17).

Of all products (*N* = 38, see Box 1 for the definition), only four were included in the 2012 National Formulary,^47^ that is, registered in the country by 2012: two of them were antimalarials from India, one AX from France, and one PC from Senegal. At the time of the purchase, all samples had more than 6 months remaining shelf life and 78.0% more than 24 months. For a same product, the price varied widely among wholesalers. On average, the AL pediatric powders were the most expensive (2.2 €/bottle), followed by AX (0.9 €/bottle, 125 mg/5 mL dosage; 1.5 €/bottle, 250 mg/5 mL dosage) and PC (0.1 €/10 tablets).

### Visual inspection.

Only items for which more than 10% of the samples did not meet the acceptance criteria for “physical appearance” and “labeling” are presented. The proportions are weighted, thus reflecting the distribution in the market.

#### Physical appearance.

**Figure 1.**
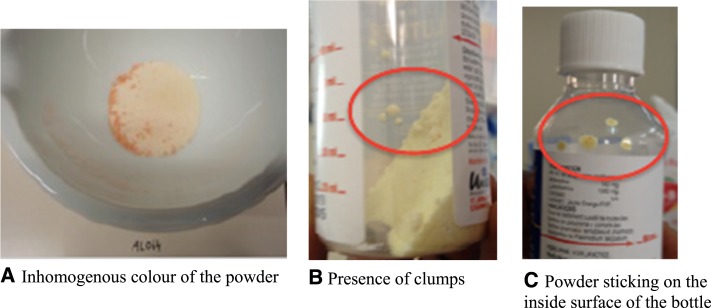
Typical nonconformities found in the physical appearance of powders for suspension of artemether/lumefantrine–containing samples.

The highest proportion of nonconformities was found for AL: 23.2% contained clumps, 22.0% were sticky, and 13.3% had heterogeneous color (Figure 1). Of the 72 nonconformities in AL samples, 50 concerned 37/49 (75.5%) samples that also failed chemical analyses. For PC, 19.2% samples presented residual powder in the blister or had sticky aspect. A relatively high proportion of powders for suspension lacked a security seal on the screw top: 87.1% of AL samples and 96.5% of AX samples. In addition, 66% of AX samples lacked a measuring cap or a spoon to administer the medicine.

#### Labeling on the outer and inner packaging.

**Figure 2.**
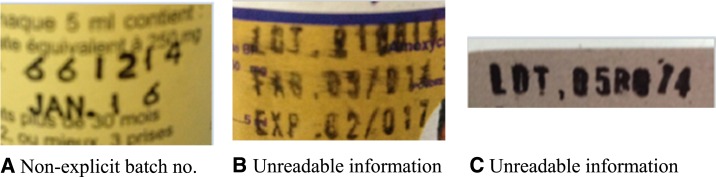
Typical nonconformities found in the labeling of samples of amoxicillin powders for suspension.

On the outer packaging, the license owner details were often missing (PC, 99.8%; AL, 82.6%; and AX, 66.4%). In addition, 67.9% of the AX samples lacked the license number (PC, 26.9%). The manufacturer details were missing for 63.6% samples of AX, 24.8% of PC and 21.8% of AL. Furthermore, concerning AX, the batch number was not clearly identified on the inner packaging of 10.1% of samples (Figure 2A) and it was unreadable for 39% of samples, mainly because of poor printing quality (Figure 2B and C).

### Chemical analysis.

**Figure 3.**
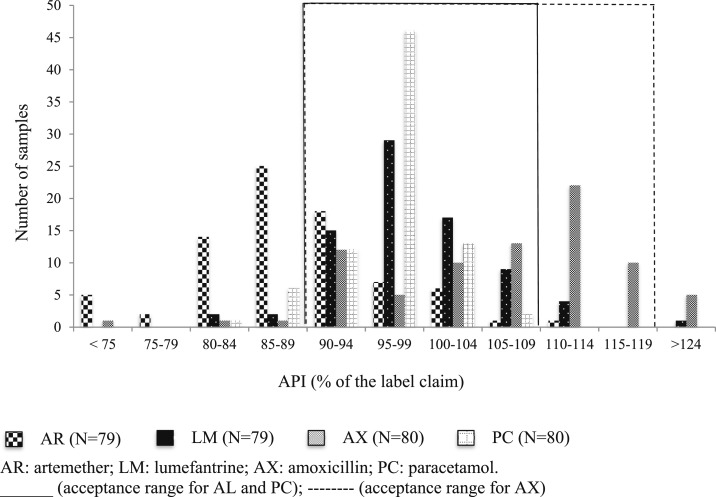
Distribution of the percent content of active ingredients vs. the stated content (*N* = 318).

All tested samples contained the stated (correct) active pharmaceutical ingredient (API). Nonetheless, 65/239 (27.2%) had at least one nonconform result. The proportions of nonconformities varied greatly across attributes and medicine type (Figure 3; Table 3), and the most frequent nonconformity was the incorrect *content in active ingredient* (26.7%). In particular, 49/79 of AL samples had an incorrect content in active ingredient (62.0%, 95% CI: 50.7, 72.2) and three had *incorrect deliverable volume*, 8/80 AX samples had an *incorrect content in active ingredient* and one had *incorrect pH* (11.3%, 95% CI: 5.9, 20.5), and 7/80 samples of PC tablets had an *incorrect content in active ingredient* (8.8%, 95% CI: 4.2, 17.2) and two failed the *mass uniformity tests* (2.5%).

**Table 3:** Numbers and percentage (%) of out-of-specifications samples by active ingredient and totals

	Artemether/lumefantrine	Amoxicillin	Paracetamol	Total
*N*/total	49/79	9/80	7/80	65/239
Crude % (C.I.)	62.0 (50.7–72.2)	11.3 (5.9–20.5)	8.8 (4.2–17.5)	27.2 (21.9–33.2)
Weighted % (C.I.)	40.0 (14.8–71.9)	4.4 (2.0–9.2)	0.3 (0.04–2.0)	1.3 (0.4–4.8)
Attributes	*N* failures (%)			
Identity	0 (0.0)	0 (0.0)	0 (0.0)	0 (0.0)
Dosage content	49 (62.0)	8 (10.0)	7 (8.7)	64 (26.7)
	47 (59.5) artemether	n.a.	n.a.	–
	10 (12.7) lumefantrine	n.a.	n.a.	–
Deliverable volume	3 (3.8)	0 (0.0)	n.a.	3 (1.9)
pH	n.a.	1 (1.3)	n.a.	1 (1.3)
Mass uniformity	n.a.	n.a.	2 (2.5)	2 (2.5)

n.a. = not applicable.

More in details, most nonconformities were due to *underdosing* (56/239), 46/49 (93.8%) nonconform AL samples contained less than 90% of the stated content in artemether, and four also less than 90% of the stated content in lumefantrine; 7/7 (100%) nonconform PC samples contained less than 90% of the stated content in PC (Table 3). Overall, the mean content in active ingredient in underdosed samples was of 83.4% (artemether), 85.6% (lumefantrine), 77.6% (AX), and 87.0% (PC).

Nonconform AL samples were found for 11/12 manufacturers. For a same product, different batch numbers were concerned. Of nine nonconform AX samples, three were *underdosed* and were manufactured with different batch numbers by the same company in DRC, whereas five were *overdosed* and were manufactured in France. All the nonconform PC samples (seven) were manufactured by the same company in DRC, with five different batch numbers. When the results were weighted by the volumes of distribution, the proportion of poor-quality samples lowered for AL (40.0% versus 62.0%), AX (4.4% versus 11.3%), and PC (0.3% versus 8.8%), suggesting that the nonconform samples may have smaller market shares.

### Risk factors for poor quality.

Four factors were investigated for correlation with poor quality: 1) the stated country of origin, 2) the inclusion in the 2012 National Formulary (i.e., marketing authorization by 2012), 3) the remaining shelf life at the time of collection, and 4) the unit price. Antimalarial samples were more likely to be of poor-quality if manufactured in DRC (DRC: 83.3%, *P* = 0.021; not defined 86.4%, *P* = 0.022) (Table 4) or if they were included in the 2012 National Formulary (86.9%, *P* = 0.009, the last finding is biased by the small sample size because only 2/12 AL products were registered in the DRC by 2012). For PC samples, poor quality was associated with domestic production (4.8%, *P* = 0.000) and to a remaining shelf life of more than 26 months (1.3%, *P* = 0.044) (the last finding is biased because all nonconform samples are manufactured by the same company and carry the same expiry date). Interestingly, no correlation was found between poor quality and the unit price.

**Table 4:** Number and weighted percentage (%) of out-of-specifications by active ingredient and risk factor

	Artemether/lumefantrine	Amoxicillin	Paracetamol
*N*/total	49/79	9/80	7/80
Stated country of origin	*N*/total (%)		
India	25/42 (34.4)	–	0/7 (0.0)
Democratic Republic of Congo	10/11 (83.3)^1^	4/14 (8.9)	7/21 (4.8)^2^
not-defined (n.d.)	13/14 (86.4)^3^	1/32 (2.1)	0/23 (0.0)
China	0/1 (0.0)	–	0/27 (0.0)
The Netherlands	1/11 (6.1)^4^	–	–
Morocco	–	–	0/1 (0.0)
France	–	4/34 (5.6)	–
Senegal	–	–	0/1 (0.0)
Registered	*N*/total (%)		
Yes	10/12 (86.9)^5^	0/7 (0.0)	0/1 (0.0)
No	39/67 (35.1)	9/73 (4.6)	7/79 (0.3)
Remaining shelf life	*N*/total (%)		
≤ 18 months	24/43 (53.6)	–	–
> 18 months	25/36 (31.9)	–	–
≤ 26 months	–	4/45 (3.5)	0/40 (0.0)
> 26 months	–	5/35 (5.0)	7/40 (1.3)^6^
Unit price (€)	*N*/total (%)		
≤ 1.6	24/40 (24.6)	–	–
> 1.6	25/39 (61.9)	–	–
≤ 0.4	–	4/40 (3.7)	–
> 0.4	–	5/40 (5.7)	–
≤ 0.0009	–	–	5/40 (0.3)
> 0.0009	–	–	2/40 (0)

*F* test, *P* ≤ 0.05 (^1^ *P* = 0.021; ^2^ *P* = 0.000; ^3^ *P* = 0.022; ^4^ *P* = 0.026; ^5^ *P* = 0.009; ^6^ *P* = 0.044).

## DISCUSSION

The chemical analysis could not screen for degradation, but no samples were expired at the time of the purchase and analysis. The chemical analysis could also not screen for falsification. However, all the samples contained the stated active ingredients; the high-performance liquid chromatography analyses did not reveal suspect peaks and the visual inspection did not find gross signs of mislabeling. This means that we did not find any suggestion of “*falsified*” medical products, that is, products that “deliberately or fraudulently misrepresent their identity, composition, or source.” Therefore, we tentatively classified the poor-quality samples as *substandards*, that is, medicines that are “authorized by national regulatory authorities but fail to meet either national or international quality standards or specifications, or in some cases, both.” Noteworthy, according to the new WHO definitions of poor-quality medicines that were approved by the World Health Assembly on May 29, 2017, a part of our samples were not registered in DRC, so they should now be classified as “*unregistered or unlicensed medical products*,” that is, medicines that “have not been assessed or approved by the relevant national or regional regulatory authority for the market in which they are marketed, distributed, or used.”

The overall proportion of poor-quality medicines was 27.2%, with great differences among target products. The highest rate concerned AL powders (62.0%). Noteworthy, the prevalence of substandard antimalarials in other surveys in sub-Saharan Africa was of 35.4% in Ghana,^48^ 27.7% in Nigeria,^49^ and 12% in Tanzania.^50^ Significantly higher rates were found in Malawi (88.4%).^51^ Comparisons need careful interpretation; however, the proportion of poor-quality AL in our study is high, even when the results are weighted (40.0%).^16,17^ The leading cause of poor-quality was the underdosing of the active ingredients (86.1%) especially of artemether.

When looking at the price of the active ingredients, that is, artemether (210 $/kg), lumefantrine (55 $/kg), AX (38 $/kg), and PC (22 $/kg),‡ we found that the higher the price, the higher the frequency of underdosed samples. Moreover, the content of active ingredients in antimalarials varied greatly among units of the same sample so that analyses had to be repeated three to four times (results not shown). This intra-batch variability was reported in other studies^51–53^ and is likely to be due to poor manufacturing practices (Box 2).^54^ Locally manufactured AL-containing samples were significantly more likely to be substandards. This may be related to the lack of adequate expertise, skills, and manufacturing equipment in DRC (Box 2).

Box 2 **Causes of poor-quality pharmaceutical products related to manufacturing practices**

1. A fixed dose combination (FDC) is a formulation of two or more active pharmaceutical ingredients (APIs) combined in a single dosage form, in fixed ratio of doses. Manufacturing technologies for FDCs are more complex than for mono-products.^65^ Combination of APIs with very different dosages, granulometry, and density, such as artemether and lumefantrine, requires proper validation of the powders mixing stage. Furthermore, improper mixing with a wide variety of inactive ingredients, such as in powders for suspension, may lead to nonhomogeneous dispersion of the APIs in the finished product, thus jeopardizing their availability in proper amounts.

2. Poor manufacturing, including lack of proper validation of processes can be sources of substandards:

 the manufacturing of a batch by mixing the remaining of the production of other batches over several days;

 the mixing of small batches (sub-batches) production to manufacture a bigger batch;

 the manufacturing of one batch by mixing active or inactive ingredients from different sources without proper validation.

 the use of inappropriate and variable-quality excipients in the production of sub-batches, leads to inter-variability in the content’s results between bottles of the same batch.

3. The determination of the dosage content of artemether/lumefantrine (AL) is intrinsically difficult. The absence of chromophore for artemether, the difference in content proportion of artemether vs. lumefantrine (weight ratio of 1/6) as well as the polarity difference between the molecules, require the availability of HPLC instruments and reagents, trained analysts and imply high operating costs.^66^ These are often not available in limited resources settings and many manufacturers lack adequate analytical methods to quantify simultaneously and accurately both artemether and lumefantrine. Thus, the quality of AL containing products is often assessed by testing only the presence of lumefantrine (non-separative method), obtaining partial results that cannot control the quality of the finished product.

Overall, our results suggest that poor-quality products may be more frequent among pediatric antimalarials than among AX- and PC-containing products, that Good Manufacturing Practices may be insufficiently implemented in local production in DRC, and that substandard products may have lower market shares than those meeting quality requirements. Interestingly, nonconformities in the physical appearance were to some extent predictive of failures in chemical analyses, especially for AL samples. We observed a high degree of shortcomings in the labeling (which is of outmost importance to trace the origin of products).^55^ In particular, the information about the manufacturer or the license’s owner was often lacking. Erasable or unidentifiable batch numbers were also observed in 10% of the AX samples.

The visual inspection also revealed problems that can lead to irrational use of medicines: most of AX powders for suspensions were sold without an adequate dosing tool, which can cause incorrect dosing, in turn contributing to antibiotic resistances, treatment failure, or toxicity. On a similar note, the use of split PC adult tablets instead of a pediatric formulation may lead to inappropriate dosing,^56^ despite the apparent cost savings advantage.^57^

To our knowledge, this is the first survey inspired by the MEDQUARG recommendations (which further informed the WHO guidelines on the conduct of survey for the quality of medicines)^58^ in DRC. Samples have been collected upstream in the distribution chain; thus, the proportion of substandard pediatric antimalarials could be higher in pharmacies, informal outlets, and in the rural regions of DRC. Weighing the results by the volumes of distribution highlighted that despite the high prevalence of substandards antimalarials in our sample, these could hold a smaller market share compared with good-quality products. Secondary findings worth of notice are as follows:

1. The incompleteness of quality requirements for packaging in the national legislation;
2. The very low rate of registered products (4/38, taking the year 2012 as reference);
3. The need of guidance, for future surveys, on how to classify samples that are at the same time “*unregistered or unlicensed*” and out-of-specifications, in light of the new WHO definitions.

The survey has some major limitations. First, it targeted a very specific segment of DRC pharmaceutical market, and its results cannot be extrapolated to the whole country. Second, the volumes of distribution still contain a degree of uncertainty. Third, some critical chemical analyses (i.e., the dissolution and/or disintegration of tablets)^59^ could not be carried out.

Despite these limitations, our findings support the hypothesis that in DRC, poor domestic manufacturing practices might be a primary cause of substandard medicines (Box 2). Our findings also suggest that visual inspection techniques may be a predictor of substandard medicines, that there is no link between poor quality and lower prices, and that adequate dosing tools are too often neglected in pediatric formulations, although they represent an essential attribute of quality.

In conclusion, there is an urgent need to strengthen the regulatory capacity in DRC, as well as to upgrade the technical skills and expertise of domestic manufacturers, to prevent the production and distribution of substandard medicines and/or unregistered medicines. Such capacity building should cover the broad range of quality assurance activities, without neglecting simple measures that may allow a first screening of poor-quality medicines, for example, visual inspection. Particular focus should be put on the quality assurance of medicines for children. Further surveys may help to orient the policies and practices of the DPM and they should, as we did in this case, be carried out in partnership with them.

## Footnotes

† The USP-Medicines Compendium was discontinued on March 2015.

‡ Information about the prices of active ingredients were obtained in the beginning of 2015 from consultants, expert in the field of the manufacturing of APIs.
